# Transgelin is a poor prognostic factor associated with advanced colorectal cancer (CRC) stage promoting tumor growth and migration in a TGFβ-dependent manner

**DOI:** 10.1038/s41419-020-2529-6

**Published:** 2020-05-11

**Authors:** Mona Elsafadi, Muthurangan Manikandan, Sami Almalki, Amer Mahmood, Tasneem Shinwari, Radhakrishnan Vishnubalaji, Mohammad Mobarak, Musaad Alfayez, Abdullah Aldahmash, Moustapha Kassem, Nehad M. Alajez

**Affiliations:** 1 Stem Cell Unit, Department of Anatomy, College of Medicine, King Saud University, Riyadh, 11461 Kingdom of Saudi Arabia; 2 Cancer Research Center, Qatar Biomedical Research Institute (QBRI), Hamad Bin Khalifa University (HBKU), Qatar Foundation (QF), Doha, Qatar; 3 Department of Histopathology, College of Medicine, King Saud University, Riyadh, 11461 Saudi Arabia; 4 Molecular Endocrinology Unit (KMEB), Department of Endocrinology, University Hospital of Odense and University of Southern Denmark, Odense, Denmark

**Keywords:** Colon cancer, Cell growth

## Abstract

Colorectal cancer (CRC) is the fourth most common cancer type globally. Investigating the signaling pathways that maintain cancer cell phenotype can identify new biomarkers for targeted therapy. Aberrant transforming growth factor-β (TGFβ) signaling has been implicated in CRC progression, however, the exact mechanism by which TGFβ exerts its function is still being unraveled. Herein, we investigated TAGLN expression, prognostic value, and its regulation by TGFβ in CRC. While TAGLN was generally found to be downregulated in CRC, elevated expression of TAGLN was associated with advanced CRC stage and predicted poor overall survival (hazard ratio (HR) = 1.8, log-rank test *P*-value = 0.014) and disease-free survival (HR = 1.6, log-rank test *P*-value = 0.046), hence implicating TAGLN as poor prognostic factor in CRC. Forced expression of TAGLN was associated with enhanced CRC cell proliferation, clonogenic growth, cell migration and in vivo tumor formation in immunocompromised mice, while targeted depletion of TAGLN exhibited opposing biological effects. Global gene expression profiling of TAGLN-overexpressing or TAGLN-deficient CRC cell lines revealed deregulation of multiple cancer-related genes and signaling pathways. Transmission electron microscopy (TEM) revealed ultrastructural changes due to loss of TAGLN, including disruption of actin cytoskeleton organization and aberrant actin filament distribution. Hierarchical clustering, principle component, and ingenuity pathway analyses revealed distinct molecular profile associated with TAGLN^high^ CRC patients with remarkable activation of a number of mechanistic networks, including SMARCA4, TGFβ1, and P38 MAPK. The P38 MAPK was the top predicted upstream regulator network promoting cell movement through regulation of several intermediate molecules, including TGFβ1. Concordantly, functional categories associated with cellular movement and angiogenesis were also enriched in TAGLN^high^ CRC, supporting a model for the molecular mechanisms linking TGFβ-induced upregulation of TAGLN and CRC tumor progression and suggesting TAGLN as potential prognostic marker associated with advanced CRC pathological stage.

## Introduction

Colorectal cancer (CRC) is one of the most common malignancies worldwide^1^. In addition to conventional histopathological phenotyping, recent years have witnessed increased interest in utilizing genetic and molecular signatures as prognostic and predictive biomarkers to identify patients at high risk of progression and recurrence^2,3^. Over the past years, molecular profiling led to the identification of a number of signaling pathways associated with CRC, where targeting such pathways demonstrated increased clinical efficacy^4,5^.

The TGFβ family, including TGFβ1, TGFβ2 and TGFβ3, is a regulator of several biological processes such as cell differentiation, cell proliferation, apoptosis, angiogenesis, and immune response^6^. Canonical TGFβ signaling involves the phosphorylation of R-Smads (SMAD2 and SMAD3) to form a complex with SMAD4 and its translocation into the nucleus, followed by induction of TGFβ-responsive genes^7^. The role of TGFβ1, which is ubiquitously expressed, in cancer development and progression has been extensively studied. Alterations in TGFβ signaling has been observed in several types of cancers, such as breast, lung, bladder, and CRC^8–11^. In CRC, TGFβ signaling exhibits contradicting effect either as a suppressor or as a stimulator, based on the cancer stage. During early stages, TGFβ plays an inhibitory role on tumor cell growth and proliferation by inducing CDK inhibitors (p15 and p21) and suppressing cell cycle stimulators such as Myc, CDK4, and CDC25A^12,13^. However, enhanced TGFβ signaling is associated with advanced CRC, low survival rate, and higher risk of recurrence^14–16^.

TAGLN, also named SM22, is a TGFβ-inducible gene highly expressed in many tissues such as bladder, prostate, stomach, colon, and uterus^17,18^. TAGLN, a member of the calponin family, is an actin-binding/gelling protein that is localized in the cytoplasm and expressed by many different types of cells, including fibroblasts, endothelial, immune, and smooth muscle cells^19^. As an actin crosslinking protein, TAGLN participates in the process of cell mobility by enhancing the formation of podosomes^20^, and in several biological processes related to cancer progression such as proliferation, differentiation, migration, invasion, and apoptosis^21^. TAGLN-deficient mice are fertile and develop normally, but they show reduced smooth muscle contractility, and pronounced changes in actin filament distribution and cytoskeletal organization^22,23^. Similar to TGFβ, TAGLN expression and function in cancer biology are dependent on the type and stage of cancer, hence the role of TAGLN in CRC biology is controversial^24–27^.

We recently reported TAGLN to play a role in skeletal stem cell proliferation, differentiation, and motility through regulation of actin cytoskeletal organization^28^. In current study, we investigated the role of TGFβ-mediated TAGLN expression in the proliferation, migration, and clonogenic potential of CRC cells in vitro and tumor formation in vivo. Transmission electron microscopy (TEM) revealed remarkable ultrastructural alterations in CRC cells with elevated and downregulated TAGLN expression. In addition, we dissected the molecular mechanism of TAGLN function using transcriptome and pathway analyses. Ingenuity pathway analysis revealed enriched functional categories and regulatory networks associated with cell migration, invasion, angiogenesis, and P38 MAPK signaling in CRC tissue exhibiting elevated TAGLN expression.

## Materials and methods

### Cell culture

HCT116 and RKO human colorectal cell lines were purchased from ATCC (Manassas, VA, USA). The HT-29 cell line was purchased from CLS Cell Lines Service (CLS Cell Lines Service GmbH, Eppelheim, Germany). Cells were maintained in Dulbecco’s Modified Eagle Medium (DMEM) containing d-glucose 4500 mg/L, 110 mg/L sodium pyruvate, 4 mM l-glutamine, and supplemented with 10% fetal bovine serum (FBS), non-essential amino acids (Gibco-Invitrogen, USA), and 1x penicillin–streptomycin (Pen–strep).

### Small-interfering RNA (siRNA) transfection

HT-29 and RKO cell lines were chosen for TAGLN knockdown because they exhibited higher TAGLN expression level (Fig. 2a). Cells were transfected with Silencer Select TAGLN-siRNA (25 nM; Ambion, USA) using Lipofectamine RNAiMAX reverse-transfection approach (Invitrogen, CA, USA) as per the manufacturer’s recommendations.

**Fig. 1:**
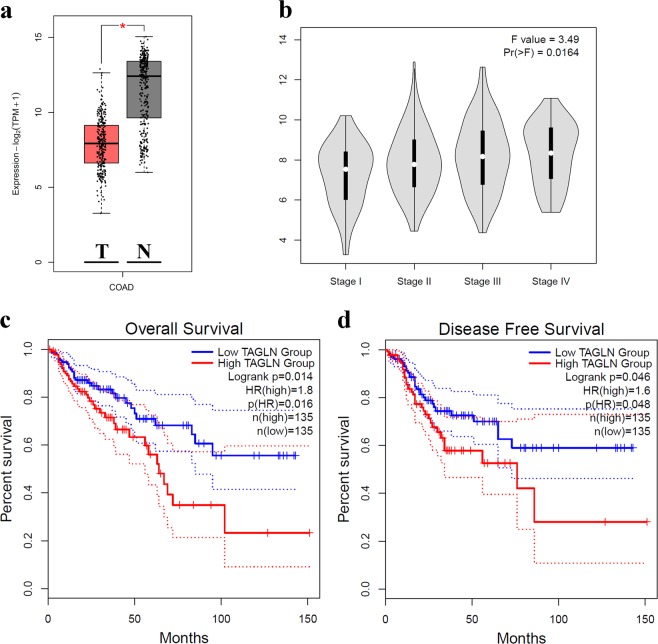
TAGLN expression is associated with advanced tumor stage and predicts worse clinical outcome. **a** Boxplot comparing the expression of TAGLN in a cohort of colon adenocarcinoma (COAD), *n* = 275 compared to normal colon tissue (*n* = 349) from the TCGA and GTEx data sets. **b** Stage plot demonstrating TAGLN expression as a function of pathological stage in COAD. Kaplan–Meier overall survival (OS, **c**) and disease-free survival (DFS, **d**) as function of median TAGLN expression in COAD.

### Establishment of the TAGLN overexpressing HCT116 cells

HCT116 cell line was chosen for TAGLN overexpression experiments because it exhibited lower basal TAGLN expression. HCT116 overexpressing TAGLN were created using lentiviral transduction approach as we previously described^29^. Lentifect™ control lentiviral particles (LP-FLUC-LV105-0205) and particles encoding for human TAGLN (LP-G0046-Lv213-200) were obtained from Genecopoeia (Genecopoeia Inc., Rockville, MD, USA). Hundred thousand cells were seeded in DMEM and 48 h later, media was removed and 20 μL of lentiviral particles in 500 μL of DMEM + 5% heat-inactivated serum and 1% Pen–Strep supplemented with 8 μg/mL polybrene (Sigma) were added to the cells. Seventy-two hours later, transduced cells were passaged and were subjected to puromycin selection (1.5 μg/mL, Sigma) to generate stable clones.

### Cell proliferation assay

Alamar Blue assay was used to assess cell viability as per the manufacturer’s recommendations (AbD Serotec, Raleigh, NC, USA) as we described before^30^. Briefly, 10 μL of alamarBlue was added to each well followed by 1 h incubation in the dark at 37 °C. Fluorescence was then measured using BioTek Synergy II microplate reader (BioTek Inc., Winooski, VT, USA) at Ex 530 nm/Em 590 nm.

### Scratch assay

In vitro wound-healing assay was conducted as described before^28^. Briefly, 0.2 × 10^6^ cells/dish were plated in a 30-mm cell culture dish. When confluent, straight line (scratch) was created using a p10 pipette tip. Time-lapse microscopy was conducted using EVOS FL Auto Cell Imaging System (ThermoFisher Scientific Life Sciences). Images were taken every 30 min over 4 days.

### Transwell migration assay

After 24 h incubation in 1% FBS-DMEM, cell migration was assessed using 8 µm pore size BD transwell migration system as described before^31^. Inserts were placed in a 12-well plate, and 60 × 10^3^ cells in 500 μL of 1% FBS-DMEM were added to the upper chamber and 1.5 μL of either 1% FBS-DMEM as a control or 10% FBS-DMEM as chemoattractant were added to the bottom chamber. Seventy-two hours later, cells on the upper surface of inserts were scraped using a cotton swap. Subsequently, the cells were fixed and stained with SIEMENNS DIFF-QUICK stain set (Siemens Healthcare Diagnostics). Stained inserts were cut and mounted on microscope slides.

### Colony-forming assay

Colony formation assay was conducted as described before^32^. Cells were seeded in a 12-well tissue culture plate at different serial dilutions (1:2 to 1:64) with an initial density of 0.015 × 10^6^ cells in each well and incubated at 37 °C under 5% CO_2_ for 6 days. Cells were allowed to grow, and on day 6, they were fixed with 70% ethanol and stained with Diff-Quik Stain Set (Siemens Healthcare Diagnostics, Inc., Newark, DE, USA). Images of colonies formed in each well were taken using a Carl Zeiss-Axio Observer 1 microscope equipped with an Axiocam MRc5 digital camera (Carl Zeiss, Oberkochen, Germany) at 10x and 20x magnification.

### Western blotting

Whole-cell lysates were prepared using RIPA buffer (ThermoFisher Scientific) followed by immunoblotting using TAGLN-specific antibody (ThermoFisher Scientific, CAT# PA5-29767, 1:5000 dilution). B-actin was used as loading control (Sigma, A3854, 1:10,000 dilution). Horseradish peroxidase-conjugated secondary antibodies (Santa-Cruz Biotechnology) and the Clarity western ECL substrate (Bio-Rad) were used for signal using C-Digit Blot Scanner (LI-COR).

### Transmission electron microscopy (TEM)

Cells under different treatment conditions were trypsinized and were subsequently washed using PBS followed by resuspending the pellets in 2.5% glutaraldehyde fixative (Electron Microscopy Sciences, cat. no. 16500) in 0.1 M phosphate buffer (pH 7.2) and kept at 4 °C for 4 h. Next, the cells were washed in 0.1 M phosphate buffer (pH 7.2) and then transferred to 1% osmium tetroxide (OsO4) in 0.1 M phosphate buffer (pH 7.2). Subsequently, cells were dehydrated using increasing grades of ethanol (10%, 30%, 50%, 70%, 90%, and 100%) for 15 min each, and then were resuspended in acetone for 15 min. The cells were then aliquoted into BEEM® embedding capsules and were infiltrated with an acetone-resin mixture. Polymerization of the resin was conducted using 70 °C oven for 12 h. In all, 0.5 μm thickness sections were cut and were stained with 1% Toluidine Blue. Subsequently, ultrathin sections (~70-nm thickness) were cut and were fixed on copper grids followed by contrasting using uranyl acetate (saturated ethanol solution) for 30 min, rinsed, and then contrasted using Reynold’s lead citrate for 5 min, followed by final rinse using distilled water. The contrasted ultrathin sections were imaged using TEM (Jeol 1010, Jeol, Tokyo, Japan).

### In vivo tumor formation assay

Animal experiments were approved by the appropriate animal care committee. Cells were harvested via trypsinization, washed in PBS, and resuspended in PBS. Approximately 1 × 10^6^ cells were suspended in 100 μL PBS per each implant and subcutaneously injected into the right flank of 6 − 8-week-old female nude mice, as described previously^33^. Tumor volumes were measured every 2–3 days using a caliper. Tumor volumes were calculated using formula (tumor length × width^2^)/2. At the end of the experiment (after 6 weeks), remaining tumors were removed, fixed in 10 % formalin, embedded in paraffin, and were subsequently sectioned and stained with hematoxylin and eosin.

### Quantitative reverse-transcription PCR (qRT-PCR)

The PureLink kit (Ambion by Life Technologies, USA, Cat No: 12183018A) was used for total RNA extraction as per the manufacturer protocol. Nanodrop spectrophotometer (Nanodrop 2000, Thermo Scientific, USA) was used for total RNA was quantification. The High Capacity cDNA Reverse-Transcription kit (Applied Biosystem, USA) was used to generate complementary DNA (cDNA) from 1 μg of the RNA using the Labnet Multigene thermocycler. Relative expression levels of selected transcripts were measured using real-time PCR (Applied Biosystem-Real-Time PCR Detection System) and the Power SYBR Green PCR master mix (Applied Biosystem, UK) or with the TaqMan Universal Master Mix II, no UNG (Applied Biosystem, USA) according to the manufacturer’s instructions. Differential expression was measured using a comparative Ct method and GAPDH as reference gene.

### Gene expression profiling

Total RNA was extracted using the PureLink RNA mini kit (Ambion by Life Technologies, USA, Cat No: 12183018A). One-hundred and fifty nanograms of total RNA were initially labeled and then hybridized to the Agilent Human SurePrint G3 Human GE 8 × 60 k microarray chip (Agilent Technologies). Microarray experiments were conducted at the Microarray Facility (Stem Cell Unit, King Saud University College of Medicine). GeneSpring GX software (Agilent Technologies) was used for normalization and data analyses. The Single Experiment Pathway analysis feature in GeneSpring 12.0 (Agilent Technologies) was used for pathway analysis. Twofold cutoff with *P* < 0.02 were used.

### Retrieval of the Cancer Genome Atlas (TCGA) colon adenocarcinoma (COAD) expression data, and gene set enrichment and modeling of gene interactions networks

COAD TCGA expression data were retrieved from the cBioPortal for Cancer Genomics (https://www.cbioportal.org/) database as we described before^34^. TCGA COAD data were stratified into TAGLN^high^ and TAGLN^low^ based on median TAGLN expression. Hierarchal and PCA analyses were conducted as we descried before^34^. Differentially expressed genes in the TAGLN^high^ vs TAGLN^low^ COAD were imported into the ingenuity pathways analysis (IPA) software (Ingenuity Systems; www.ingenuity.com/) followed by functional annotations and regulatory network analysis using upstream regulator analysis (URA), downstream effects analysis (DEA), and mechanistic networks (MN) prediction algorithms. *Z*-score was used to determine association significance.

### TAGLN expression and survival analysis in the TCGA/GTEx dataset

The expression of TAGLN in the TCGA/GTEx dataset and its association with tumor stage, overall and disease-free survival was conducted using the GEPIA2 database (http://gepia2.cancer-pku.cn) as described before^35^.

### Statistical analysis

All results are presented as the mean ± standard deviation (SD) from at least three independent experiments and *P*-values < 0.05 (using unpaired two-tailed *t*-test) were considered significant. Pearson’s correlation was conducted using Graphpad prism 6.0.

## Results

### TAGLN expression is associated with advanced tumor stage and predicts worse clinical outcome

First, we explored the expression of TAGLN in 275 tumor and 349 normal tissue from the TCGA/GTEx COAD dataset. Interestingly, TAGLN was downregulated in COAD compared to normal colon tissue (Fig. 1a), which is concordant with our previously published work^30^. Notably, TAGLN expression was elevated during stage progression (Fig. 1b). Additionally, high TAGLN expression was associated with worse overall survival (OS, hazard ratio (HR) = 1.8, log rank *P* = 0.014, Fig. 1c) and worse disease-free survival (DFS, HR = 1.6, log rank *P* = 0.046, Fig. 1d). Therefore, we postulated loss of TAGLN during early stage and subsequent re-acquisition of TAGLN expression in late stages of COAD, suggesting a possible role for TAGLN in driving colon cancer progression.

### TAGLN enhances CRC cell proliferation and colony formation

**Fig. 2:**
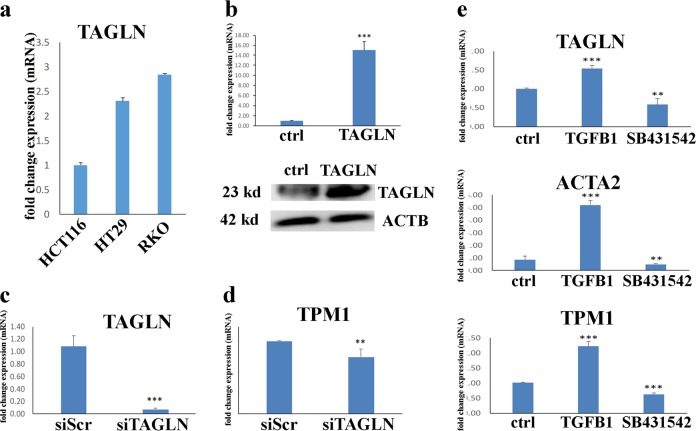
TAGLN is a TGFβ responsive gene in CRC. **a** TAGLN mRNA expression in three different CRC cell lines based on the CCLE database: HCT116, HT-29, and RKO. Expression level is compared to TAGLN expression in HCT116 cells. **b** qRT-PCR of TAGLN expression in HCT116 control cells or cells transduced with TAGLN-overexpressing lentiviral vector (upper panel). Western blotting for TAGLN (upper panel) in HCT116-TAGLN vs. control cells. β-Actin was used as the loading control. QRT-PCR for TAGLN (**c**) or TPM1 (**d**) gene expression 3 days post transfection of RKO cells with TAGLN-siRNA (siTAGLN) or scrambled-siRNA (siScr). Data are presented as fold change in mRNA expresion. **e** qRT-PCR of TAGLN, TPM1, and ACTA2 gene expression in RKO cells treated with TGFβ1 (10 ng/µl) or SB431542 (10 µM), compared to control cells. The two-tailed *t*-test was used to compare different treatment groups. **P < 0.005, ***P < 0.0005.

To gain better understanding of the role of TAGLN in in CRC biology, we established an HCT116 CRC cell line stably overexpressing TAGLN (HCT116-TAGLN) utilizing lentiviral delivery system. HCT116 cells were chosen because they exhibited low-basal TAGLN expression (Fig. 2a). Overexpression of TAGLN in HCT116-TAGLN cells was confirmed by the presence of high levels of the TAGLN mRNA transcript (Fig. 2b, upper panel) and protein (Fig. 2b, lower panel). Additionally, we performed siRNA-mediated loss-of-function of TAGLN expression in the RKO CRC line, which has higher basal TAGLN expression (Fig. 2a). siTAGLN transfection led to substantial reduction in TAGLN expression in the RKO model (Fig. 2c). Similarly, TPM1, a previously characterized TGFβ-responsive gene, was also downregulated in TAGLN-depleted RKO cells (Fig. 2d). We recently reported that TAGLN is a TGF*β* –responsive gene in bone marrow stromal stem cells^28^. Thus, we examined the effect of exogenous TGF*β* treatment on TAGLN expression, as well as on the expression of a number of TGF*β*-responsive genes (ACTA2, and TPM1), in the RKO colon cancer cell model. Exposing RKO cells to TGF*β*1 (10 ng/mL) enhanced TAGLN, ACTA2, and TMP1 mRNA expression (Fig. 2e). In contrast, inhibition of TGF*β*1 signaling using type I activin receptor-like kinase (ALK) inhibitor, SB431542 (10 μm), resulted in downregulation of TAGLN, ACTA2, and TPM1 (Fig. 2e).

**Fig. 3:**
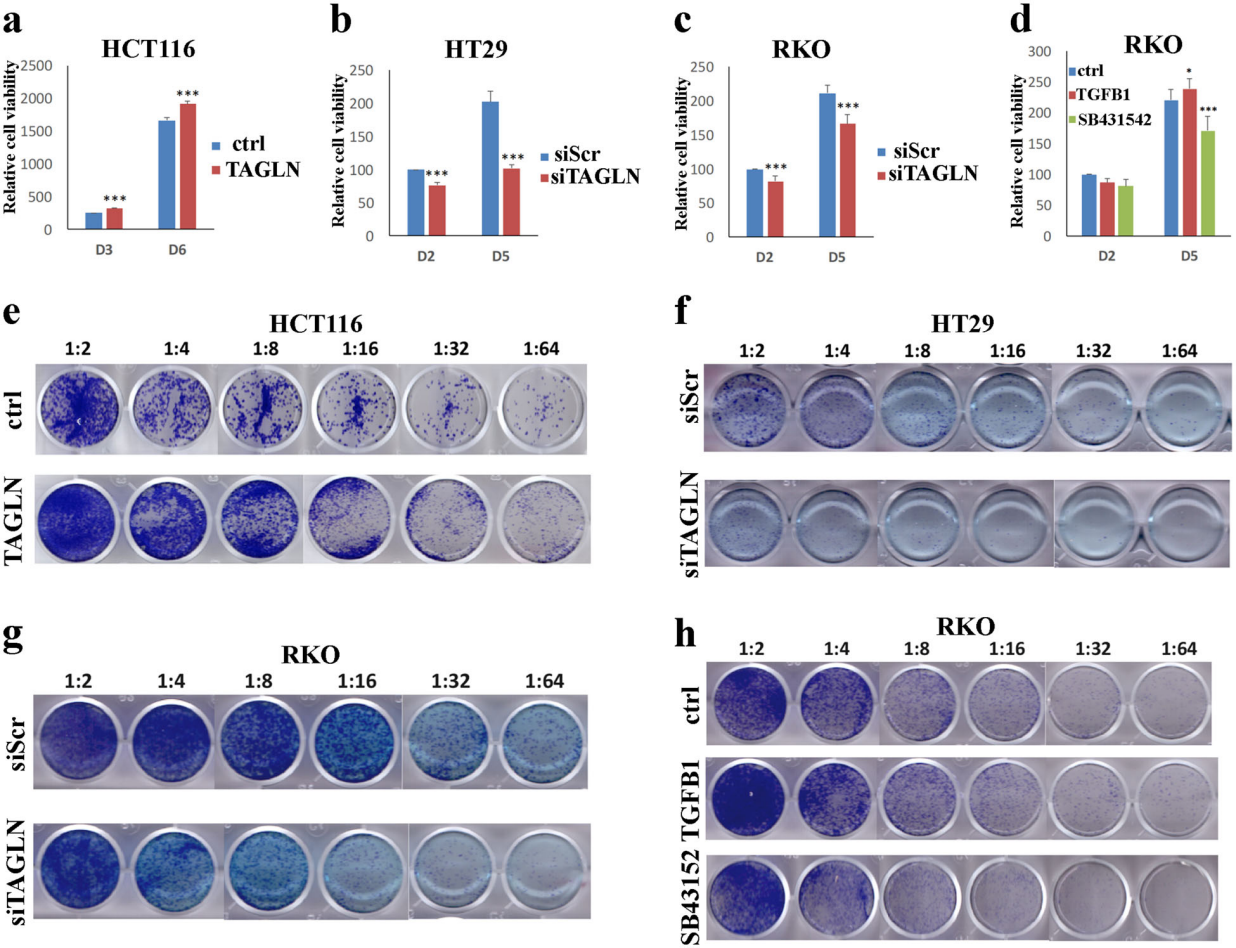
TAGLN induces CRC cell proliferation and colony formation. Alamar blue assay showing cell viability in HCT116 overexpressing TAGLN compared to control cells (**a**) and in TAGLN-depleted HT-29 (**b**) or RKO (**c**) cells at the indicated time points. **d** Effect of exogenous TGFβ (10 ng/mL) and TGFβ inhibition using SB431542 (10 µM) on RKO cell viability. Data are shown as mean ± S.D. of at least two independent experiments. *P < 0.05, ***P < 0.0005. **e** Representative clonogenic assay showing clonogenicity of HCT116 cells overexpressing TAGLN or TAGLN-depleted HT-29 (**f**) and RKO (**g**) cells. **h** Effects of TGFβ (10 ng/mL) and TGFβ inhibition using SB431542 (10 µM) on RKO colony formation ability. Plates were stained with Diff-Quik stain set on day 6. Wells are representative of at least two independent experiments for each condition.

We subsequently investigated the biological ramifications of TAGLN overexpression or knockdown on CRC cells using cell viability and colony formation unit (CFU) assays. TAGLN-HCT116 exhibited significant increase in cell proliferation and colony formation ability (Fig. 3a, e). In contrast, downregulation of TAGLN expression was associated with reduced cell proliferation and colony formation employing the HT-29 (Fig. 3b, f) and RKO (Fig. 3c, g) cell models. Similarly, activation or inhibition of TGFβ signaling exhibited similar biological effects on the RKO cell model (Fig. 3d, h). Taken together, our data suggests a role for TAGLN in promoting CRC proliferation and colony formation.

### TAGLN enhances CRC migration and in vivo tumor formation

**Fig. 4:**
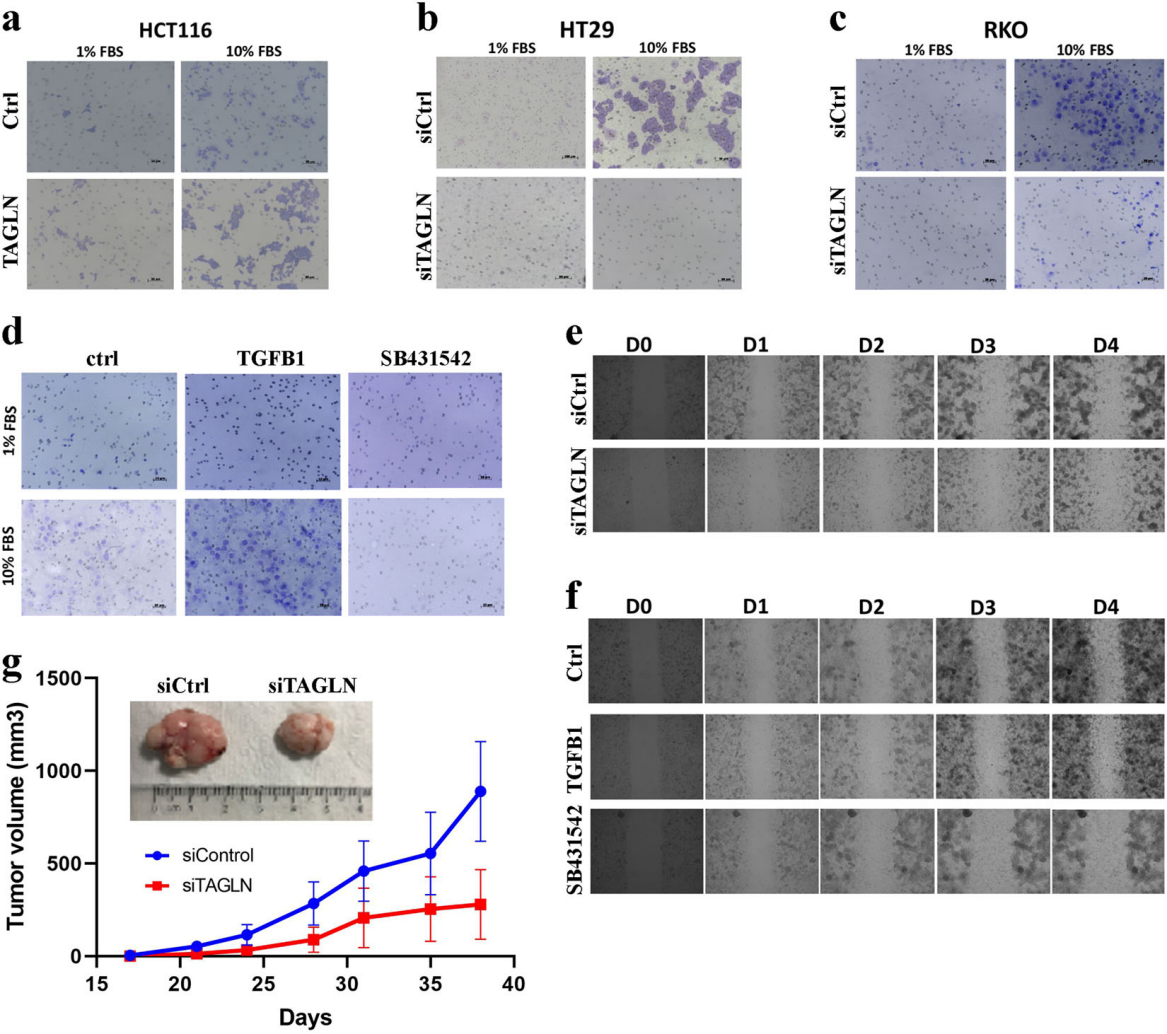
TAGLN promotes CRC cell migration and in vivo tumor formation. **a** Transwell migration assay showing increase of cell migration in HCT116 overexpressing TAGLN in response to 10% FBS attractant. Effects of TAGLN depletion on HT-29 (**b**) and RKO (**c**) cell migration using transwell migration system. **d** Effect of exogenous TGFβ (10 ng/mL) and TGFβ inhibition using SB431542 (10 µM) on RKO cell migration using the transwell migration system. Effects of TAGLN depletion (**e**) and exogenous TGFβ (10 ng/mL) and TGFβ inhibition using SB431542 (10 µM) (**f**) on RKO cell migration using wound-healing assay. Time-lapse microscopy was conducted using EVOS FL Auto Cell Imaging System where images were taken every 30 min over 4 days. **g** Subcutaneous tumor formation of control (siControl) and TAGLN-depleted (siTAGLN) RKO cells in nude mice. Data are presented as mean (tumor volume) ± S.E., *n* = 5 per group. Representative tumors at the end of experiment is shown (upper panel).

The effects of TAGLN on CRC cell migration was subsequently investigated using transwell migration assay. HCT116 cells overexpressing TAGLN exhibited enhanced migration capabilities (Fig. 4a), whereas TAGLN-depleted HT-29 (Fig. 4b) and RKO (Fig. 4c) cells exhibited reduced cell migration. In agreement with those data, RKO cells treated with TGFβ1 (10 ng/µL) exhibited enhanced cell migration (Fig. 3d), whereas inhibition of TGFβ signaling using SB431542 (10 µM) reduced RKO cells migration potential (Fig. 4d). Similar effects of TAGLN depletion, exogenous TGFβ treatment, and TGFβ inhibition using SB431542 was observed using wound-healing assay (Fig. 4e, f). Additionally, TAGLN-depleted RKO cells exhibited reduced tumor formation in vivo (Fig. 4g), corroborating the in vitro results, thus highlighting an important role for TAGLN in driving CRC migration and tumor formation.

### TAGLN regulates several functional categories and signaling pathways in CRC

**Fig. 5:**
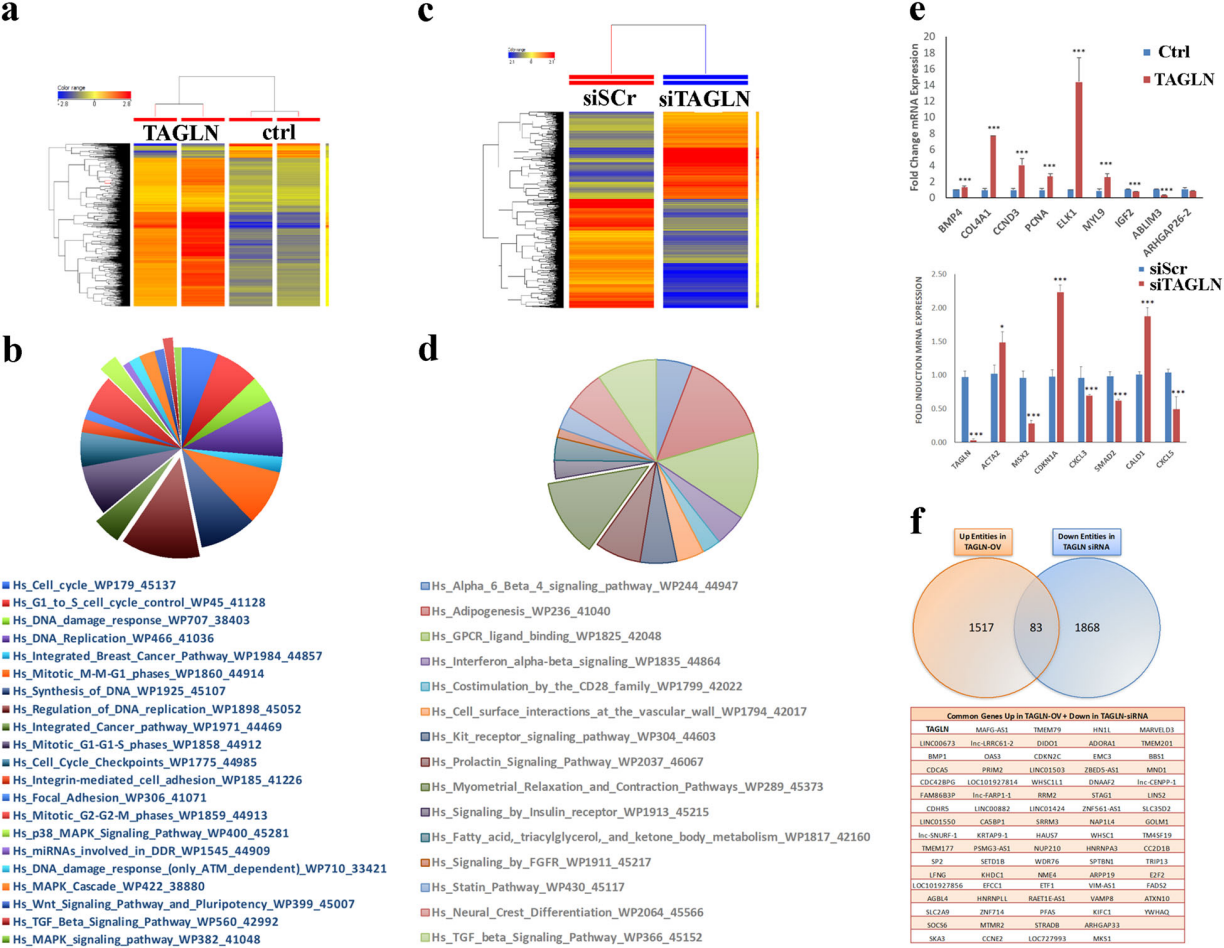
TAGLN regulates several functional categories and signaling pathways in CRC. **a** Hierarchical clustering of TAGLN-overexpressing or control HCT116 cells based on differentially expressed mRNA levels. Each row represents one replica sample and each column represents an mRNA. Expression level of each gene in a single sample is depicted according to the color scale. **b** Pie chart illustrating the distribution of the top pathway designations for the upregulated genes in TAGLN-overexpressing HCT116 cells. **c** Hierarchical clustering of TAGLN-depleted (siTAGLN) and control (siScr) RKO cells based on differentially expressed mRNA levels. **d** Pie chart illustrating the distribution of the top pathway designations for the downregulated genes in siTAGLN transfected RKO cells. **e** qRT-PCR validation of selected genes from microarray, *n* = 2, *P < 0.05, **P < 0.005, ***P < 0.005. **f** Venn-diagram depicting the overlap between the upregulated genes in TAGLN-overexpressing HCT116 and the downregulated genes in TAGLN-depleted RKO cells.

To unravel the molecular mechanism underlying the biological role of TAGLN in CRC, we performed transcriptome analysis on HCT116 cells overexpressing TAGLN, as well as on TAGLN-depleted RKO cells. Hierarchical clustering based on differentially expressed mRNAs revealed separation between HCT116 cells overexpressing TAGLN and control cells (Fig. 5a and Supplementary Table 1). Top affected pathways in HCT116 overexpressing TAGLN are illustrated as pie chart (Fig. 5b). Similar changes were also observed in TAGLN-depleted RKO cells (Fig. 5c, d and Supplementary Table 2). Validation of selected number of genes from the microarray data is shown in Fig. 5e. We subsequently crossed the two data sets and identified 83 common genes that were upregulated in HCT116-TAGLN and were downregulated in siTAGLN-RKO cells (Fig. 5f).

### TGFβ-mediated TAGLN expression induces ultrastructure changes in CRC cells

**Fig. 6:**
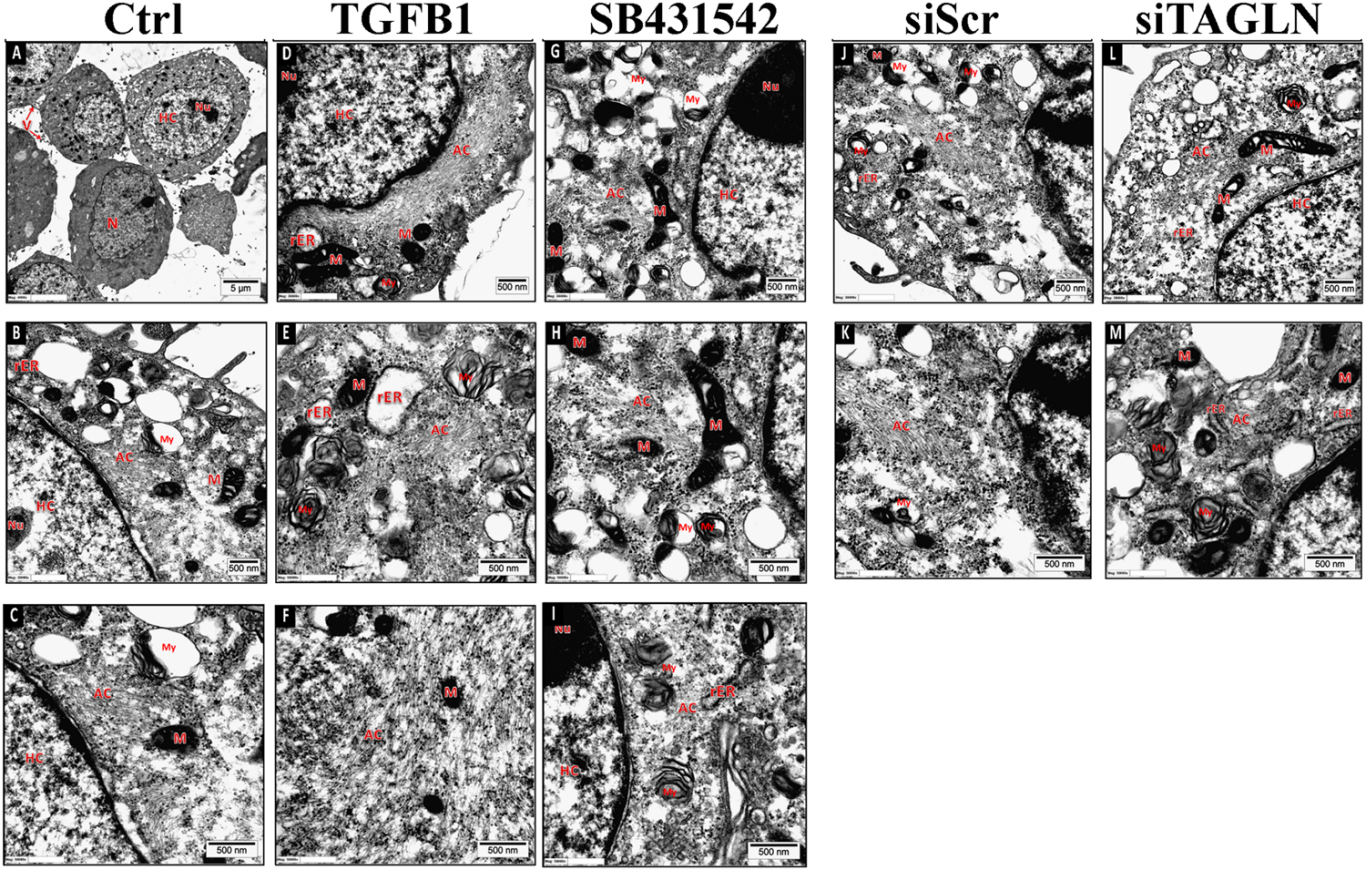
Transmission electron microscopy (TEM) illustrating the ultrastructural characteristics of RKO cells under different treatment conditions. RKO cells were treated with exogenous TGFβ (10 ng/mL), TGFβ inhibition using SB431542 (10 µM), or TAGLN depletion using siTAGLN. N nucleus, Nu nucleolus, AC actin filaments, V microvilli, M mitochondria, rER rough endoplasmic reticulum, V microvilli, HC heterochromatin, AC actin filaments, my myelin figure.

Given its role as an actin-binding protein, we investigated the effects of TGFβ and its inhibitor, SB431542, as well as TAGLN depletion on actin cytoskeleton changes, and actin microfilaments polymerization in RKO cells. TAGLN perturbation using SB431542 (Fig. 6g, h, i) or through siRNA-mediated knockdown (Fig. 6l, m) revealed significant inhibition of actin microfilament polymerization. In contrast, exogenous TGFβ1 treatment led to prominent actin filaments organized as bundles/aggregates distributed in the whole cytoplasm and in peri-nuclear locations (Fig. 6d–f). Moreover, rough endoplasmic reticulum (RER) was cystically dilated in TGFβ1-treated cells, suggesting increased protein synthesis activity (Fig. 6e).

### TAGLN expression predicts more aggressive phenotype in patients with COAD

**Fig. 7:**
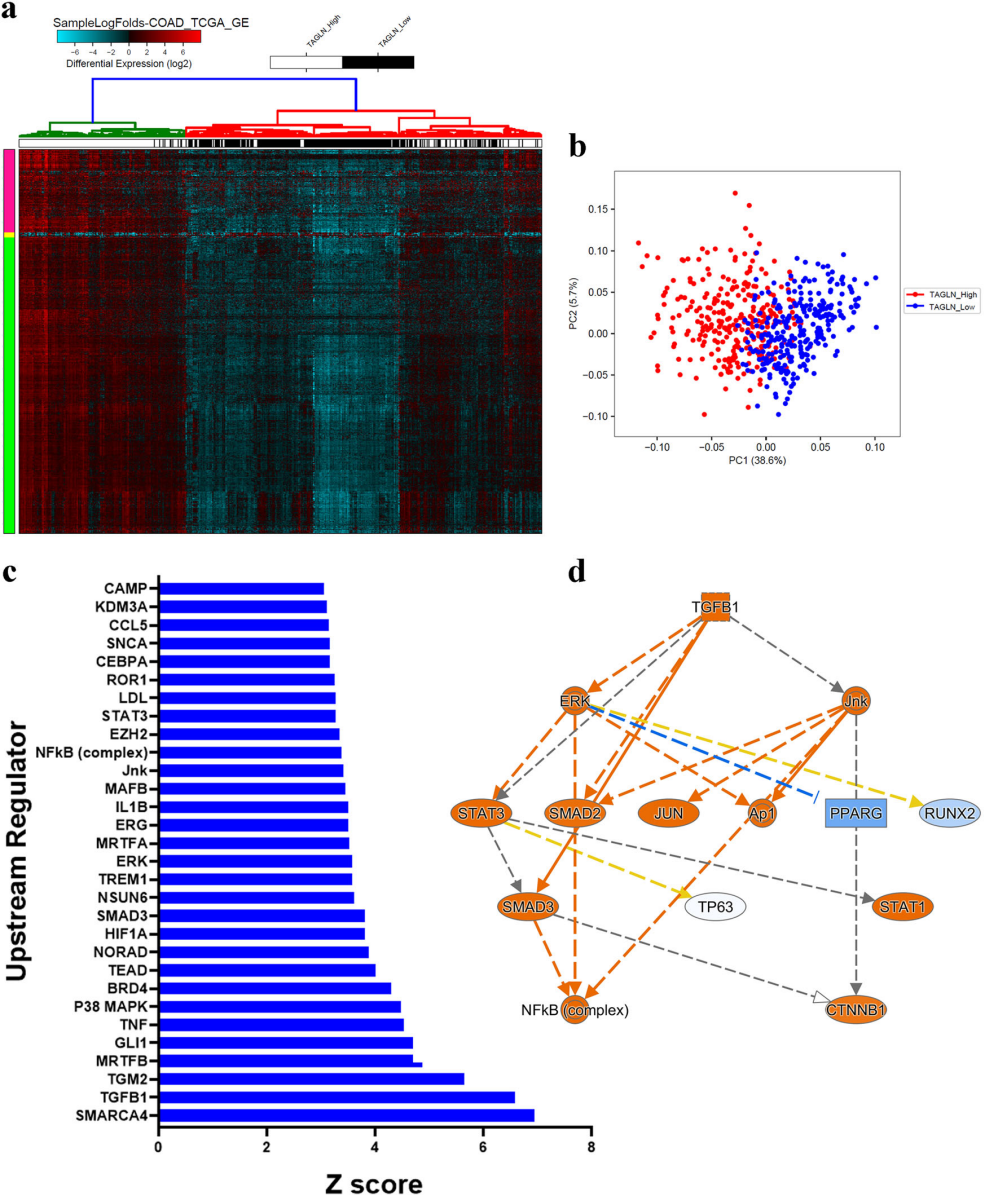
Molecular signature of TAGLN^high^ COAD. **a** Heat map clustering of TAGLN^high^ (*m* = 296) and TAGLN^low^ (*n* = 296) COAD. Samples were stratified into TAGLN^high^ and TAGLN^low^ based on median TAGLN expression. Each row represents expression level of an mRNA (log2). mRNA expression level in a single sample is depicted according to the color scale. **b** Principal component analysis (PCA) for TAGLN^high^ (*m* = 296) and TAGLN^low^ COAD. **c** Bar-graph depicting the top thirty significantly activated upstream regulator networks in TAGLN^high^ vs TAGLN^low^ COAD. Activation *Z*-score is indicated on the *x*-axis. **d** Illustration of the TGFβ1 mechanistic network.

Our in vitro and in vivo data demonstrated a role for TAGLN in promoting CRC in vitro and in vivo, however, whether such effects for TAGLN can be recapitulated in patients with COAD was not clear. Therefore, the expression data from 592 patients with COAD from the TCGA dataset was retrieved and patients were stratified into TAGLN^high^ and TAGLN^low^. Hierarchical clustering and principle component analysis (PCA) revealed separation of the TAGLN^high^ and TAGLN^low^ groups, suggesting tangible differences between the two tumor groups at the molecular level (Fig. 7a, b, and Supplementary Table 3). IPA analysis on the differentially expressed genes between TAGLN^high^ and TAGLN^low^ groups highlighted remarkable activation of a number of mechanistic networks, including SMARCA4, TGFβ1, and P38 MAPK (Fig. 7c and Supplementary Table 4). Illustration of the TGFβ1 mechanistic network in shown in Fig. 6d. Those data are in agreement with our in vitro data connecting TGFβ and TAGLN in CRC.

**Fig. 8:**
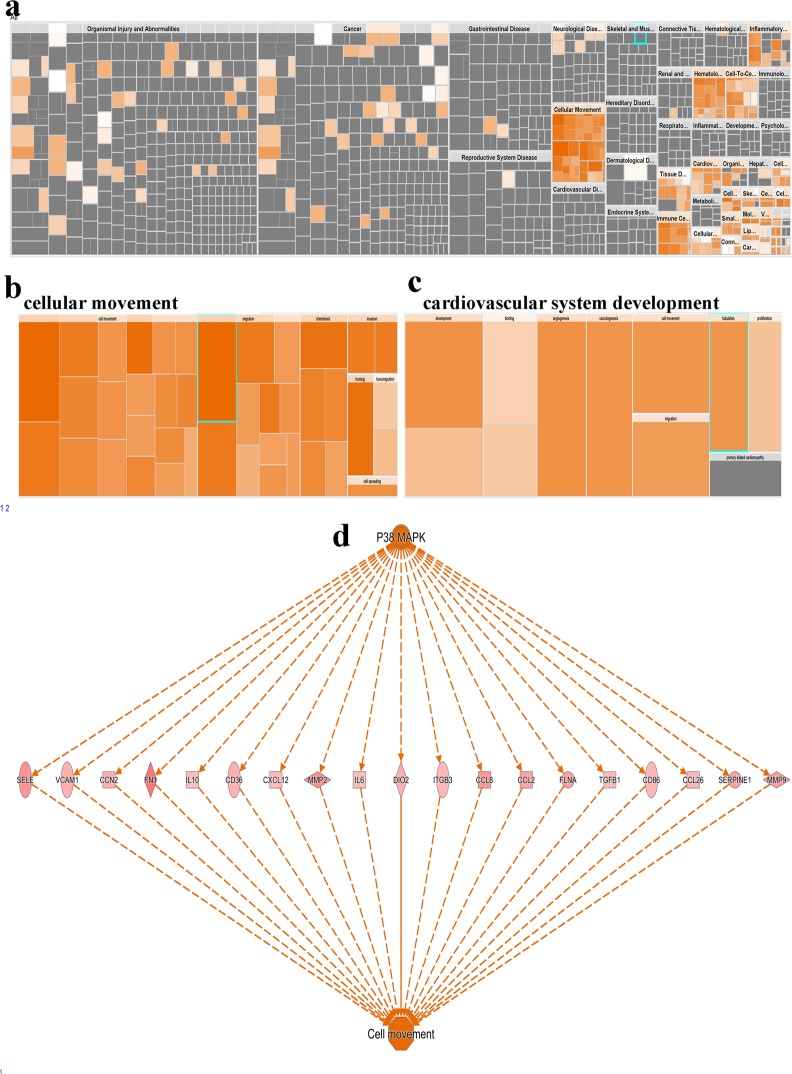
TAGLN^high^ COAD is enriched in functional categories promoting cell migration, angiogenesis, and P38 MAPK signaling. **a** Disease and function heat map depicting enrichment in the indicated functional and disease categories in the differentially expressed genes in TAGLN^high^ vs. TAGLN^low^ COAD patients’ data based on IPA analysis. Heat map-illustrating enrichment in cellular movement (**b**) and cardiovascular system development (**c**) functional categories. **d** Illustration of the predicted P38 MAPK regulator network and its predicted effects of cell movement.

IPA analysis additionally revealed remarkable enrichment in a number of functional categories in TAGLN^high^ COAD, predominantly functional categories associated with cellular movement and angiogenesis (Fig. 8a–c and Supplementary Table 5). Interestingly, P38 MAPK was the top predicted regulator promoting cell movement through regulation of several intermediate molecules, including TGFβ1, MMP2, MMP9, and CXCL12 (Fig. 8d).

## Discussion

Increased risk of poor prognosis of CRC patients is associated with intrinsic characteristics of tumor cells that include rapid proliferation, evidence of invasion and distant metastasis^36^. Suppressing cancer cell proliferation and invasion is an effective strategy to reduce cancer mortality risk and to increase survival rates. In the current study, we report a role for TAGLN, as a TGFβ responsive gene, in promoting CRC in vitro and in vivo and its association with poor clinical outcome. We suggested a model where TAGLN expression is reactivated during late stages of CRC to drive a more aggressive CRC phenotype.

A number of earlier studies highlighted TAGLN expression as a TGFβ-responsive gene in various biological systems, such as smooth muscle cells, mesenchymal stem cells, embryonic fibroblast cells, neural crest stem cells, and epithelial cells^20,28,37,38^. We have previously shown that TGFβ1-induces TAGLN expression via the SMAD2/3 signaling pathway in bone marrow MSCs^28^. Our current and previous data revealed significant downregulation of TAGLN expression in CRC compared to adjacent normal tissue^30^. Interestingly however, elevated expression of TAGLN was associated with more advanced CRC pathological stage and worse clinical outcome, suggesting a role for TAGLN in driving CRC disease progression. Concordant with our previous report, TAGLN was observed to be specifically upregulated in response to TGFβ activation in CRC, whereas TGFβ-signaling inhibitor SB431542 abolished this effect^28^.

The TGFβ signaling pathway is one of the most important intracellular signaling pathways participating in cancer development and metastasis, and is involved in the regulation of several biological processes associated with oncogenesis such as cell proliferation, differentiation, migration, and apoptosis^21^. TGFβ plays a dual function in regulating cell migration and growth. During early tumor progression, TGFβ acts as an inhibitor of proliferation and migration, whereas later during advanced CRC and metastasis, it enhances tumor growth and invasion^39,40^. Induction of the protooncogene c-fos by TGFβ1 in poorly invasive HD3 colon carcinoma cells was associated with induction of some growth-inhibitory signals, while this signal transduction pathway is suppressed in TGFβ1-stimulated invasive colon carcinoma U9^40^. Given that TAGLN expression is under the control of TGFβ, this explains the paradoxical observations of the role of TAGLN in CRC development and progression.

Although previous studies demonstrated a role of TAGLN as a tumor suppressor and its inhibition is an early event in tumor transformation and development^24,25,41^, recent clinical and functional proteomic evidence showed its association with enhanced cell invasion, cell survival, activation of epithelial-to-mesenchymal transition (EMT) and resistance to anoikis (apoptosis induced by cell detachment) during the distant metastasis stage^42^. The CRC cellular model used in our studies seems to reflect the role of TAGLN in advanced disease as it enhanced both cell proliferation and cell migration. In support of this role in CRC, TAGLN expression is upregulated in CRC tissues present in lymph node-metastasis^42^. In addition, manipulating TAGLN expression influences the expression of other metastasis-related genes and pathways^27,42^. Finally, strong association between elevated expression of TAGLN and TGFβ1 gene expression in CRC tumor samples and poor prognosis further support the association between TAGLN and advanced CRC disease.

Various actin-binding proteins and signaling pathways are implicated in regulating the dynamics of actin architecture^43^. As an actin crosslinking protein, TAGLN plays a role in actin cytoskeleton organization and is involved in supporting the cell shape and polarity, along with various cellular and biological processes such as proliferation, migration, apoptosis, and differentiation^21^. Our data support the role of TAGLN in maintaining ultrastructural integrity of CRC. In addition, the ability of TAGLN to enhance cell migration is supportive of its effects on cellular cytoskeleton. During cell motility, the actin cytoskeleton regulates protrusion formation, distribution of contractile filaments and assembly/disassembly of focal adhesion molecules through changes in the actin dynamic assembly and disassembly^43^. Several studies have reported inhibition in cell migration and adhesion due to disruption of actin cytoskeleton organization^44,45^. In addition to its possible direct role on actin cytoskeleton organization, TGFβ-mediated TAGLN expression is associated with upregulation of a number of additional TGFβ-responsive genes such as alpha-smooth muscle actin (ACTA2) and tropomyosin (TPM1), which are involved in cell motility and stabilization of cytoskeleton actin filaments and were reported to be regulated in metastatic cancer^46,47^. Molecular profiling comparing TAGLN^high^ and TAGLN^low^ colon cancer patients’ specimens shedding some light on the activation of a number of mechanistic networks, including SMARCA4, TGFβ1, and P38 MAPK while remarkable enrichment in a number of functional categories associated with cellular movement and angiogenesis was most prominent in TAGLN^high^, suggesting biological relevance to our in vitro findings in patients with colorectal cancer.

## Conclusion

Our data describe a model for the molecular mechanisms linking TGFβ-induced upregulation of TAGLN and CRC tumor progression. In addition, the clinical data suggest that TAGLN expression is a possible prognostic marker for advanced CRC disease, hence implicating TAGLN as poor prognostic factor offering possible therapeutic opportunity for CRC.

## Supplementary information

Supplementary table 1

Supplementary table 2

Supplementary table 3

Supplementary table 4

Supplementary table 5

## References

[1] Brenner H, Kloor M, Pox CP (2014). Colorectal cancer. Lancet.

[2] Erstad DJ, Tumusiime G, Cusack JC (2015). Prognostic and predictive biomarkers in colorectal cancer: implications for the clinical surgeon. Ann. Surgical Oncol..

[3] Alajez NM (2016). Large-scale analysis of gene expression data reveals a novel gene expression signature associated with colorectal cancer distant recurrence. PLoS ONE.

[4] Shaath, H., Toor, S. M., Nair, V. S., Elkord, E. & Alajez, N. M. Transcriptomic analyses revealed systemic alterations in gene expression in circulation and tumor microenvironment of colorectal cancer patients. *Cancers* **1**, 1–19 (2019). 10.3390/cancers11121994 PMC6966620 31835892

[5] Sanz-Garcia E, Grasselli J, Argiles G, Elez ME, Tabernero J (2016). Current and advancing treatments for metastatic colorectal cancer. Expert Opin. Biol. Ther..

[6] Massague J (2012). TGFbeta signalling in context. Nat. Rev. Mol. cell Biol..

[7] Shi Y, Massague J (2003). Mechanisms of TGF-beta signaling from cell membrane to the nucleus. Cell.

[8] Tian F (2003). Reduction in Smad2/3 signaling enhances tumorigenesis but suppresses metastasis of breast cancer cell lines. Cancer Res..

[9] Imai K (2013). Bronchioloalveolar invasion in non-small cell lung cancer is associated with expression of transforming growth factor-beta1. World J. Surgical Oncol..

[10] Fan Y (2014). TGF-beta-induced upregulation of malat1 promotes bladder cancer metastasis by associating with suz12. Clin. Cancer Res..

[11] Jung B, Staudacher JJ, Beauchamp D (2017). Transforming growth factor beta superfamily signaling in development of colorectal cancer. Gastroenterology.

[12] Derynck R, Akhurst RJ, Balmain A (2001). TGF-beta signaling in tumor suppression and cancer progression. Nat. Genet..

[13] Sheen YY, Kim MJ, Park SA, Park SY, Nam JS (2013). Targeting the transforming growth factor-beta signaling in cancer therapy. Biomol. Ther..

[14] Robson H, Anderson E, James RD, Schofield PF (1996). Transforming growth factor beta 1 expression in human colorectal tumours: an independent prognostic marker in a subgroup of poor prognosis patients. Br. J. Cancer.

[15] Tsamandas AC (2004). The potential role of TGFbeta1, TGFbeta2 and TGFbeta3 protein expression in colorectal carcinomas. Correlation with classic histopathologic factors and patient survival. Strahlenther. Onkol..

[16] Friedman E (1995). High levels of transforming growth factor beta 1 correlate with disease progression in human colon cancer. Cancer Epidemiol. Biomarkers Prev..

[17] Assinder SJ, Stanton JA, Prasad PD (2009). Transgelin: an actin-binding protein and tumour suppressor. Int. J. Biochem. Cell Biol..

[18] Dos Santos Hidalgo G, Meola J, Rosa ESJC, Paro de Paz CC, Ferriani RA (2011). TAGLN expression is deregulated in endometriosis and may be involved in cell invasion, migration, and differentiation. Fertil. Steril..

[19] Sheppard D (2006). Transforming growth factor beta: a central modulator of pulmonary and airway inflammation and fibrosis. Proc. Am. Thorac. Soc..

[20] Yu H (2008). Transgelin is a direct target of TGF-beta/Smad3-dependent epithelial cell migration in lung fibrosis. FASEB J..

[21] Dvorakova M, Nenutil R, Bouchal P (2014). Transgelins, cytoskeletal proteins implicated in different aspects of cancer development. Expert Rev. Proteom..

[22] Zhang JC (2001). Analysis of SM22alpha-deficient mice reveals unanticipated insights into smooth muscle cell differentiation and function. Mol. Cell. Biol..

[23] Zeidan A (2004). Ablation of SM22alpha decreases contractility and actin contents of mouse vascular smooth muscle. FEBS Lett..

[24] Yeo M (2010). Loss of SM22 is a characteristic signature of colon carcinogenesis and its restoration suppresses colon tumorigenicity in vivo and in vitro. Cancer.

[25] Li Q, Shi R, Wang Y, Niu X (2013). TAGLN suppresses proliferation and invasion, and induces apoptosis of colorectal carcinoma cells. Tumour Biol..

[26] Lee EK, Han GY, Park HW, Song YJ, Kim CW (2010). Transgelin promotes migration and invasion of cancer stem cells. J. Proteome Res..

[27] Zhou HM (2016). Transgelin increases metastatic potential of colorectal cancer cells in vivo and alters expression of genes involved in cell motility. BMC Cancer.

[28] Elsafadi M (2016). Transgelin is a TGFbeta-inducible gene that regulates osteoblastic and adipogenic differentiation of human skeletal stem cells through actin cytoskeleston organization. Cell Death Dis..

[29] Hamam D (2014). microRNA-320/RUNX2 axis regulates adipocytic differentiation of human mesenchymal (skeletal) stem cells. Cell Death Dis..

[30] Vishnubalaji R (2015). Genome-wide mRNA and miRNA expression profiling reveal multiple regulatory networks in colorectal cancer. Cell Death Dis..

[31] Al-toub M (2013). Pleiotropic effects of cancer cells’ secreted factors on human stromal (mesenchymal) stem cells. Stem Cell Res. Ther..

[32] Vishnubalaji R (2016). MicroRNA-320 suppresses colorectal cancer by targeting SOX4, FOXM1, and FOXQ1. Oncotarget.

[33] Vishnubalaji R (2019). Neoplastic transformation of human mesenchymal stromal cells mediated via LIN28B. Sci. Rep..

[34] Vishnubalaji R, Shaath H, Elkord E, Alajez NM (2019). Long non-coding RNA (lncRNA) transcriptional landscape in breast cancer identifies LINC01614 as non-favorable prognostic biomarker regulated by TGFbeta and focal adhesion kinase (FAK) signaling. Cell Death Discov..

[35] Tang Z (2017). GEPIA: a web server for cancer and normal gene expression profiling and interactive analyses. Nucleic Acids Res..

[36] Daly ME (2012). Orthovoltage intraoperative radiotherapy for locally advanced and recurrent colorectal cancer. Dis. Colon Rectum.

[37] Chen S, Kulik M, Lechleider RJ (2003). Smad proteins regulate transcriptional induction of the SM22alpha gene by TGF-beta. Nucleic Acids Res..

[38] Qiu P, Feng XH, Li L (2003). Interaction of Smad3 and SRF-associated complex mediates TGF-beta1 signals to regulate SM22 transcription during myofibroblast differentiation. J. Mol. Cell. Cardiol..

[39] Engle SJ (1999). Transforming growth factor beta1 suppresses nonmetastatic colon cancer at an early stage of tumorigenesis. Cancer Res..

[40] Hsu S, Huang F, Hafez M, Winawer S, Friedman E (1994). Colon carcinoma cells switch their response to transforming growth factor beta 1 with tumor progression. Cell Growth Differ..

[41] Yeo M (2006). Loss of transgelin in repeated bouts of ulcerative colitis-induced colon carcinogenesis. Proteomics.

[42] Lin Y (2009). Association of the actin-binding protein transgelin with lymph node metastasis in human colorectal cancer. Neoplasia.

[43] Tang DD, Gerlach BD (2017). The roles and regulation of the actin cytoskeleton, intermediate filaments and microtubules in smooth muscle cell migration. Respiratory Res..

[44] Pollard TD, Cooper JA (2009). Actin, a central player in cell shape and movement. Science.

[45] Gerthoffer WT (2008). Migration of airway smooth muscle cells. Proc. Am. Thorac. Soc..

[46] Lee HW (2013). Alpha-smooth muscle actin (ACTA2) is required for metastatic potential of human lung adenocarcinoma. Clin. Cancer Res..

[47] Pawlak G (2004). Alterations in tropomyosin isoform expression in human transitional cell carcinoma of the urinary bladder. Int. J. Cancer.

